# A family of GFP-like proteins with different spectral properties in lancelet *Branchiostoma floridae*

**DOI:** 10.1186/1745-6150-3-28

**Published:** 2008-07-03

**Authors:** Diana Baumann, Malcolm Cook, Limei Ma, Arcady Mushegian, Erik Sanders, Joel Schwartz, C Ron Yu

**Affiliations:** 1 Stowers Institute for Medical Research, 1000 E 50th St., Kansas City, MO, 64110, USA; 2 Department of Microbiology, Molecular Genetics, and Immunology, University of Kansas, Kansas City, KS, 66160, USA; 3 Department of Anatomy and Cell Biology, University of Kansas Medical Center, Kansas City, KS, 66160, USA

## Abstract

**Background:**

Members of the green fluorescent protein (GFP) family share sequence similarity and the 11-stranded β-barrel fold. Fluorescence or bright coloration, observed in many members of this family, is enabled by the intrinsic properties of the polypeptide chain itself, without the requirement for cofactors. Amino acid sequence of fluorescent proteins can be altered by genetic engineering to produce variants with different spectral properties, suitable for direct visualization of molecular and cellular processes. Naturally occurring GFP-like proteins include fluorescent proteins from cnidarians of the *Hydrozoa *and *Anthozoa *classes, and from copepods of the *Pontellidae *family, as well as non-fluorescent proteins from *Anthozoa*. Recently, an mRNA encoding a fluorescent GFP-like protein AmphiGFP, related to GFP from *Pontellidae*, has been isolated from the lancelet *Branchiostoma floridae*, a cephalochordate (Deheyn *et al*., *Biol Bull*, 2007 213:95).

**Results:**

We report that the nearly-completely sequenced genome of *Branchiostoma floridae *encodes at least 12 GFP-like proteins. The evidence for expression of six of these genes can be found in the EST databases. Phylogenetic analysis suggests that a gene encoding a GFP-like protein was present in the common ancestor of *Cnidaria *and *Bilateria*. We synthesized and expressed two of the lancelet GFP-like proteins in mammalian cells and in bacteria. One protein, which we called LanFP1, exhibits bright green fluorescence in both systems. The other protein, LanFP2, is identical to AmphiGFP in amino acid sequence and is moderately fluorescent. Live imaging of the adult animals revealed bright green fluorescence at the anterior end and in the basal region of the oral cirri, as well as weaker green signals throughout the body of the animal. In addition, red fluorescence was observed in oral cirri, extending to the tips.

**Conclusion:**

GFP-like proteins may have been present in the primitive *Metazoa*. Their evolutionary history includes losses in several metazoan lineages and expansion in cephalochordates that resulted in the largest repertoire of GFP-like proteins known thus far in a single organism. Lancelet expresses several of its GFP-like proteins, which appear to have distinct spectral properties and perhaps diverse functions.

**Reviewers:**

This article was reviewed by Shamil Sunyaev, Mikhail Matz (nominated by I. King Jordan) and L. Aravind.

## Background

Genetically encoded fluorescent probes are indispensable tools for *in vivo *imaging of molecules, cells and whole organisms. Among the fluorescent proteins that have been developed as reporters, members of the GFP family, with the green fluorescent protein of hydroid *Aequorea victoria *as its founding member [1,2] are unique in that their chromophore/fluorophore is formed solely from the polypeptide chain itself, and their maturation as well as light emission does not require any cofactors other than oxygen. Members of the GFP family are found in many cnidarians, where they display a range of excitation and emission spectra, from fluorescence to bright coloration in the visible light. GFPs proved to be extraordinarily amenable to genetic manipulation: some of the useful traits of the engineered GFP derivatives include shifts in the maxima of excitation and/or emission; timed responses, such as kindling or color change after excitation; photoactivation; assembly of functional monomers from the fragments of the molecule; and others [3-5]. With all this knowledge about structure-function relationships in the GFP family, there is nonetheless a considerable interest in naturally occurring GFP-like proteins with novel properties.

The evolutionary history of the GFP family and its relationship to other proteins are not well-understood. In addition to *Cnidaria*, fluorescent proteins with significant sequence similarity to GFPs have been found several years ago in marine crustaceans of the *Pontellidae *family [6]. In an unrelated work, the structure of one domain (G2) within nidogen, a protein component of basement membranes in various groups of metazoan animals, was found to share spatial similarity with GFP and GFP-like proteins [7]. The common fold, described as 11-stranded β-barrel, is so close in nidogen G2 domain and in GFPs that the superimposition of these structures is possible with root mean square deviation of 2.5Å between 195 carbon alpha atoms (out of approximately 225 in GFP). The lack of discernible sequence similarity between G2 domains and GFPs, however, leaves open the evolutionary questions, i.e., whether the two β-barrels descend from the common ancestral gene, and, if such common ancestor existed, what was its function and in which organism did it reside.

In this work, we present the results of our analysis of a family of GFP-like proteins from a cephalochordate, the lancelet *Branchiostoma floridae*. The imaging of adult animals reveals anatomically discrete areas of green and red fluorescence surrounding the oral aperture. The nearly-completely sequenced genome of *B. floridae *encodes at least 12 GFP-like proteins, which appear to have arisen by duplication after separation of the protostome and deuterostome lineages. Several of these genes are represented in the public EST libraries obtained from eggs and from different stages of embryo development. To assess the utility of these genes as reporters, we expressed the humanized versions of two of these proteins in a mammalian system.

## Results and Discussion

We were interested in the evolutionary provenance of the β-barrel proteins and searched for the homologs of various β-barrels in the EST databases. When GFP and GFP-like proteins were used as queries in the TBLASTN searches of the non-human, non-mouse EST database at NCBI, we detected a few trivial matches with perfect identity to several cloned GFPs, apparently originating from the unfiltered fragments of the cloning vectors (data not shown). To our surprise, however, the overwhelming majority of the matches with significant sequence similarity were from the EST libraries corresponding to various developmental stages of the lancelet *B. floridae*. The lancelet ESTs represented evolutionarily distinct sequences not found in other organisms. Translations of these ESTs were 30–40% identical to copepod GFPs, their nearest database homologs, and were more distant from the cnidarian GFPs and GFP-like proteins. At this level of similarity, nonetheless, the matches had high statistical significance (E-value < 10^-10^), and the residues that are involved in the chromophore formation in other GFP-like proteins appeared to be well-conserved, suggesting that the lancelet GFP-like sequences may represent a fluorescent protein.

**Figure 1:**
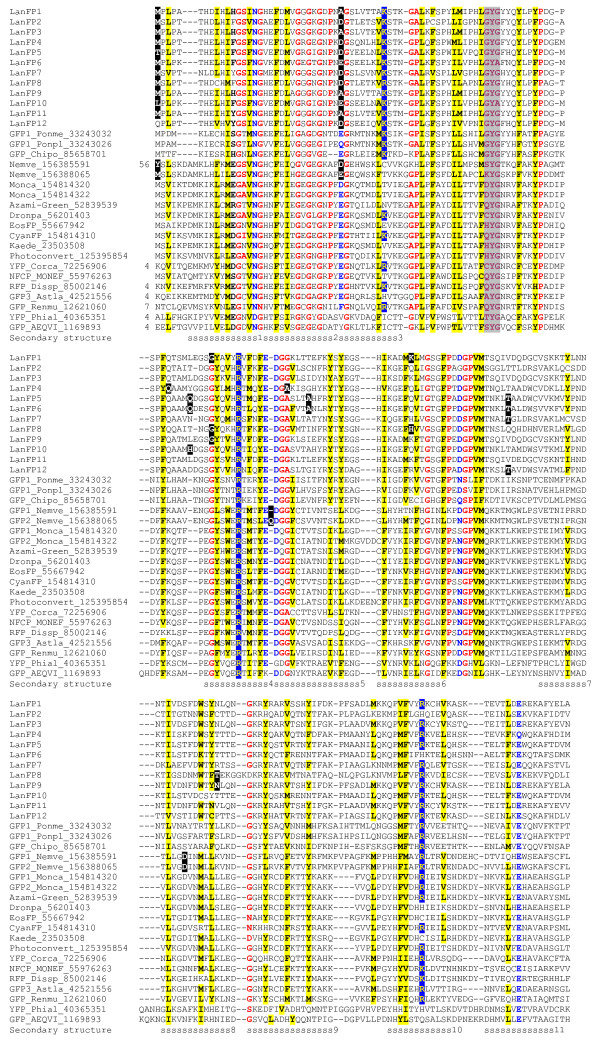
**Sequence conservation in the GFP family**. Multiple alignment of protein sequences of GFP-like proteins from cnidarians, copepods, and lancelet. Protein sequences of gene products predicted from the genome assembly of *B. floridae *were clustered at the 90% identity cutoff, and one representative per cluster that did not contain internal deletions was included into the alignment (see Table 1 for details). Identifier of each sequence in JGI genome browser or in GenBank is given after each sequence. The consensus secondary structure derived from multiple known three-dimensional structures of GFP-like proteins is shown below the alignment. Red type indicates conserved small or kinky side chains (G, S, A, or P), yellow shading indicates conserved bulky hydrophobic residues (I, L, V, M, F, Y, or W), blue type indicates conserved acidic or amidic residues (D, E, N, or Q), blue shading indicates conserved basic residues (K or R), purple type with gray shading indicates the tripeptide directly participating in rearrangement that leads to the chromophore formation, and white type on black indicates the amino acid whose codon contains an intron in the known genome sequence. Species abbreviations are as follows: Aeqvi, *Aequorea victoria*; Astla, *Astrangia lajollaensis*; Chipo, *Chiridius poppei*; Corca, *Corynactis californica*; Dissp, *Discosoma sp*. RC-2004; Monca, *Montastraea cavernosa*; Monef, *Montipora efflorescens*; Nemve, *Nematostella vectensis*; Phial, *Phialidium sp*. SL-2003; Ponpe, *Pontella meadi*; Ponpl, *Pontellina plumata*; Renmu, *Renilla muelleri*.

A more detailed analysis of the ESTs indicated that there may be several distinct, closely related members of the GFP family in lancelets, and searches in the DNA trace archive of the *B. floridae *genome suggested that it may encode additional homologs of GFPs not represented in the EST databases. Most recently, we took advantage of the DOE Joint Genome Institute release v.1.0 of the annotated genome assembly of *B. floridae *and collected a non-redundant set of genes encoding full-length homologs of GFP (Table 1 and Figure 1). In addition, we examined the first release of the genome assembly of cnidarian *Nematostella vectensis*, the only other sequenced genome that is known to encode GFP-like proteins.

**Table 1:** properties of lancelet GFP-like genes and their products.

***B. floridae *genes**	**JGI gene model ID – JGI model name**	**Comments**	**Identity to** ** other ** **LanFP** ** proteins, %**	**ESTs ** **(source tissues)**	**Identity (%) and gene ID of the best match in *B. lanceolatum*/in** **copepods**
LanFP1	7875 – FGENESH2_PG.SCAFFOLD_1000062		48–90	21 (egg, larva, neurula)	51% 169125805/32% 33243028
LanFP2	7877 – FGENESH2_PG.SCAFFOLD_1000064		51–99	33 (adult, egg, gastrula, neurula)	57% 169125805/36% 33243028
LanFP3	31376 – FGENESH2_PG.SCAFFOLD_264000004		49–61	19 (neurula, gastrula, egg, larva)	51% 169125805/34% 33243028
LanFP4	7879 – FGENESH2_PG. SCAFFOLD_1000066		51–98	15 (egg, larva)	57% 169125805/36% 33243028
LanFP5	3655 – ESTEXT_FGENESH2_PG.C_4080036	two fused GFP-like proteins?	50–54	1 (adult)	39% 33243028/76% 169125805 (84% 169125805)
LanFP6	7878 – FGENESH2_PG.SCAFFOLD_1000065		51–97	1 (larva)	54% 169125805/37% 33243028
LanFP7	21366 – ESTEXT_FGENESH2_PG.C_2370020		48–60	1 (larva)	56% 169125805/40% 33243028
LanFP8	11646 – FGENESH2_PG.SCAFFOLD_58000032		54–70		91% 169125797/40% 33243028
LanFP9	33503 -FGENESH2_PG.SCAFFOLD_549000016		49–55		52% 169125805/33% 33243028
LanFP10	11648 – FGENESH2_PG.SCAFFOLD_58000034		48–82		77% 169125805/37% 33243032
LanFP11	35422 – FGENESH2_PG.SCAFFOLD_722000001		48–56		72% 169125805/38% 33243028
LanFP12	7881 – FGENESH2_PG.SCAFFOLD_1000068		49–55		69% 169125805/30% 33243032
LanFP13	3657 – FGENESH2_PG.SCAFFOLD_408000038		50–84		79% 169125805/36% 33243034
	7876 – FGENESH2_PG.SCAFFOLD_1000063	similar to LanFP7, internal deletion			
	43701 – FGENESH2_PG.SCAFFOLD_149000048	similar to LanFP11; long unrelated N-terminal extension – probably prediction artefact			
	43778 – FGENESH2_PG.SCAFFOLD_150000025	similar to LanFP13			
	31374 -FGENESH2_PG.SCAFFOLD_264000002	similar to LanFP6; long insertion			
	31375 -FGENESH2_PG.SCAFFOLD_264000003	similar to LanFP12			
	3656- FGENESH2_PG.SCAFFOLD_408000037	similar to LanFP10			
	33504- FGENESH2_PG.SCAFFOLD_549000017	similar to LanFP4			
	11648 – FGENESH2_PG.SCAFFOLD_58000033	similar to LanFP6			
	11649 – FGENESH2_PG.SCAFFOLD_58000035	similar to LanFP7			
	11650 -FGENESH2_PG.SCAFFOLD_58000036	similar to LanFP7			
	35196 – FGENESH2_PG.SCAFFOLD_771000005	similar to LanFP4			

Comparative analysis of genomes and of protein sequences paints a picture of an ancient origin of the GFP-family proteins and their evolution by vertical descent followed by frequent gene loss (Figure 1, Table 1, and Additional file 1). The phylogenetic tree inferred from the aligned protein sequences indicates that all cnidarian GFPs form one well-supported clade in the tree, all copepod GFPs form another, and the set of lancelet GFP-like proteins forms the third clade (Additional file 1). The branching order in the midpoint-rooted tree follows the Metazoan phylogeny, with copepod and lancelet clades being closest to each other. This is compatible with the presence of an ancestral GFP in the common ancestor of Metazoa, followed by loss of this gene in some of the present-day species and lineage-specific expansion in the others.

A further indication of the ancient ancestry of GFPs in Metazoa comes from the comparison of the intron positions in cnidarian and cephalochordate genes. GFP genes in *B. floridae *and *N. vectensis *appear to share at least one intron in the homologous position of the codon 32 (Figure 1). The probability of independent insertion of an intron into a homologous site within the orthologous genes in two lineages of eukaryotes is thought to be less than 20% [8,9] and may be less than 10% within Metazoa [9], suggesting that the intron in this codon is much more likely to be ancestral than convergently inserted. The position of this conserved intron close to the 5' termini of the GFP genes is compatible with the recently documented 5'-to-3' bias towards retention of ancestral introns and the opposite bias towards intron gain and loss in multicellular organisms [10]. Moreover, another intron is present in the start codon of almost all genes in lancelet and of two genes in corals also supports this view, though the sequence conservation at the beginning of the coding region is lower and their alignment is more ambigous.

Six of the genes encoded by the genome of *B. floridae *are represented in the EST libraries made from eggs, various stages of embryo development and adult animals. We wondered whether any of these genes may encode proteins that would confer fluorescence to the animals.

Reports of yellow or yellow-green fluorescence in various tissues of lancelets, most notably in fixed neural cells, have been published in the past, but were attributed to fluorescence of small molecules, such as retinol derivatives or monoamines [11]. To get a more up-to-date picture of fluorescence of live animals, we surveyed the animals in captivity. Adult lancelets *B. floridae *gathered off the Florida coast could be housed successfully for almost a year in a salt-water aquarium. We observed typical burrowing behaviour: usually only the anterior end of the animal could be seen protruding above the surface of the substrate, but was quickly withdrawn on disturbance.

**Figure 2:**
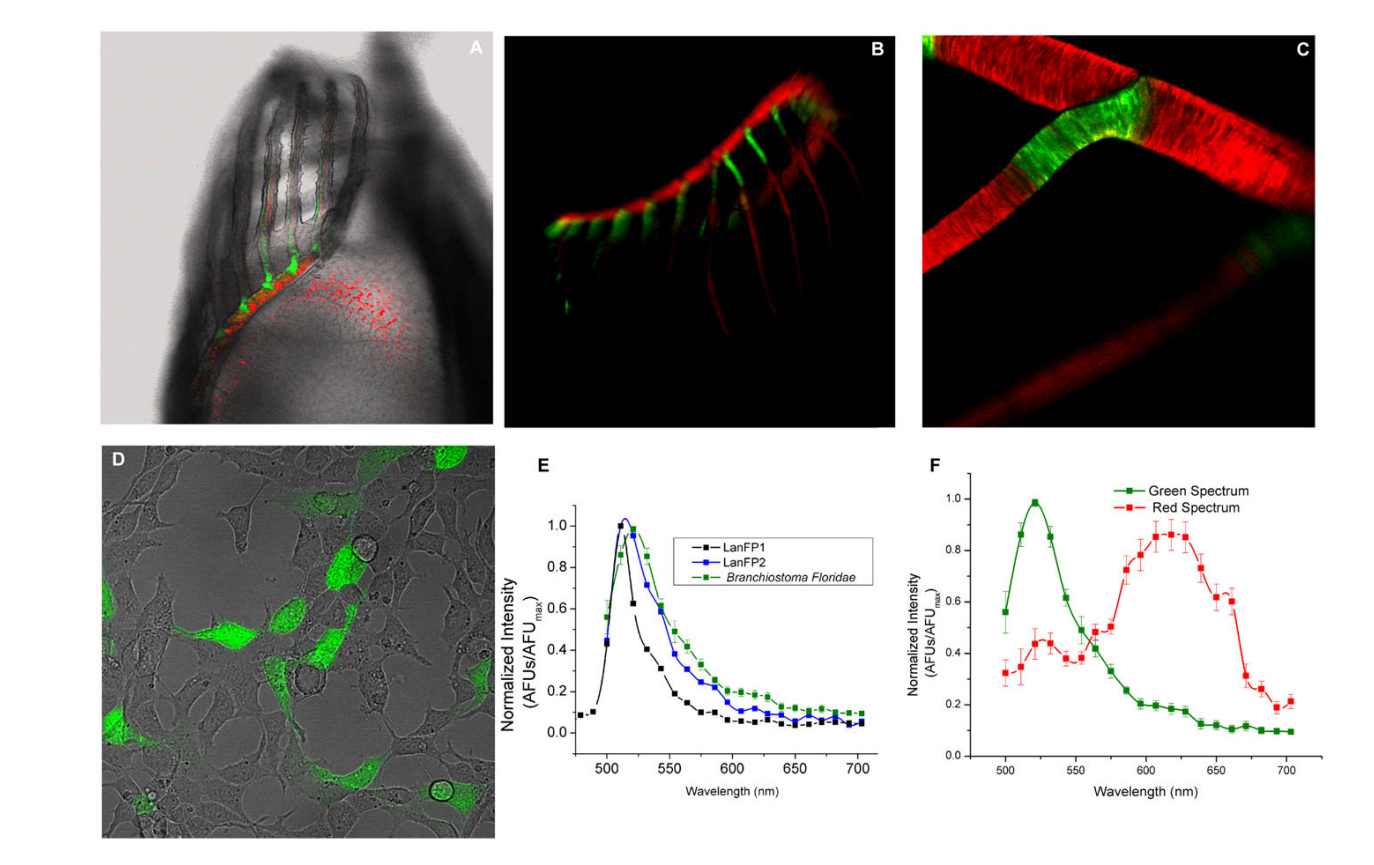
**Fluorescence of adult *B. floridana *and of heterologously expressed LanFP1 and LanFP2**. (A) Brightfield images of the oral cavity of adult *Branchiostoma floridae *are overlayed onto fluorescence images. The green and red fluorescence was separated using linear unmixing of the two most distinct fluorescence spectra. (B) The composite image of the entire oral cavity created by merging images obtained using either GFP or Texas Red filter. (C) Limited overlap between the green and red emitted fluorescence in the L-shaped rod connecting the oral cirri to the skeletal muscle (a higher-resolution fragment of panel C). (D) HEK-293 cells transiently expressing LanFP1. (E) The emission spectra from the adult *Branchiostoma floridae*. (F) Comparison of the green spectrum from panel E with the emission spectra of LanFP1 and LanFP2s expressed in HEK-293 cells.

Using both widefield and confocal fluorescence microscopy, we observed green fluorescence, distributed diffusely around most of the body but most clearly pronounced in the anterior part, in almost every animal (Figure 2A). The fluorescent signal was concentrated in the oral cirri [12], the semi-circle of tentacles that surrounds the buccal cavity of the animal (Figure 2A–C). The cirri overlay a web-like sheet of integument and specialized muscles and together with oral hood they form the pre-oral structures that feed into the long inner vestibule. The strongest green fluorescence signal was emitted by the linked L-shaped structures that appear to support each tentacle (Figure 2B–C). This fluorescence pattern corresponds to the position of cirral skeletal rods [12], and it appears that fluorescent cells are located mostly within (or sheathed around) the horizontal part and the lower portion of the vertical part of the "L" formed by each skeletal rod. These observations are in broad agreement with imaging results of Deheyn and co-authors [13], except that the bases of skeletal rods, which in their hands displayed fluorescence only in Asian species of *Branchiostoma*, are brightly fluorescent in *B. floridae *adults that we studied.

Interestingly, we also observed clear red fluorescence signal that was similarly concentrated around oral cirri, and strongly overlapped with the areas of green fluorescence (Figure 2A–C and 2E). Most of the red signal was restricted to the cirral skeletal ring and oral cirrus just above the cirral skeletal rods, but some of it extended to the distal tips of the oral cirri (Figure 2C). Analysis of full spectral information at each pixel in the image indicates that there was little pixel-to-pixel overlap between the areas associated with the green and red fluorescence, suggesting spatial segregation of red and green emitters, perhaps even cell-specific expression of each (though higher optical resolution than our 3 μm would be needed for a definitive proof of this).

In order to further study the properties of lancelet GFP-like genes and to relate them to the fluorescence observed in live animals, we have synthesized cDNAs for two of the lancelet genes that corresponded to the largest number of database ESTs, with codon frequencies optimized for expression in the mammalian system, and transfected them into HEK 293 cells (Figure 2D). Cells expressing each construct emitted green light when excited with blue light. LanFP1 and LanFP2 were also expressed in E. coli and affinity-purified: both preparations exhibited maximal absorption at 500 nm, and the emission spectra of both proteins were similar (λ_max _= 510 nm for LanFP1, and 516 nm for LanFP2). Molecular brightness of the two fluorescent proteins in transfected HEK 293 cells and in purified samples was, however, significantly different, with LanFP1 much brighter than LanFP2. Analyses of the protein preparations purified from *E. coli *showed that the quantum efficiency of LanFP1 was by the two orders of magnitude higher than that of LanFP2, and about half of the brightness of the potent Venus derivative of YFP (Additional file 2).

We exploited the fact that LanFP1 and LanFP2 have slightly different excitation and emission spectra and compared them with endogenous fluorescent signals from the cirri. The emission spectrum from *B. floridae *has a maximum at 521 nm, which is much closer to that of LanFP2-expressing HEK-293 cells and of affinity-purified LanFP2 than to the corresponding values of LanFP1 (Figure 2F).

Analysis of the EST libraries indicates that transcripts encoding both LanFP1 and LanFP2 are abundant in eggs and embryos, though thus far no matching ESTs have been found in the libraries from adult animals. We did not detect any fluorescent signals in the red spectrum in mammalian cells singly or doubly transfected with LanFP1 or LanFP2.

Days before the submission of this manuscript, 21 cDNA sequences encoding proteins of GFP family from a European species *B. lanceolatum *were released by Genbank. They apparently include naturally occurring and engineered cDNAs, most of which display either green or red fluorescence [14]. There is no one-to-one correspondence between these sequences and the products of GFP-like genes encoded by *B. floridae *genome.

## Conclusion

In this work, we identified a family of genes encoding GFP-like proteins in lancelet *B. floridae *and expressed two of them in bacterial and mammalian cell cultures. Both proteins, LanFP1 and LanFP2, exhibit green fluorescence of different brightness and distinct spectral properties when expressed in mammalian cells, and they, along with at least four other GFP-like proteins, appear to be expressed at various stages of lancelet development as judged from the analysis of the EST libraries. Moreover, adult lancelets display overlapping but distinct patterns of green and red fluorescence, with green fluorescence shifted towards more basal regions of oral cirri and red fluorescence more prominent at the cirri tips.

Recently, Deheyn and co-authors reported the sequence of LanFP2 and the pattern of green fluorescence in lancelet eggs, larvae and adults [1], and Israelsson [14] reported a similarly diverse family of cDNAs expressing red and green fluorescent proteins in *B. lanceolatus*. It remains to be investigated which of the GFP-like proteins encoded by the Florida lancelet genome are responsible for fluorescence observed by these authors and by us. Intriguingly, LanFP1 appears to be a bright GFP, whereas LanFP2 fluoresces only dimly *in vitro *and in heterologous expression systems. At the same time, its emission spectrum is the closest match to the emission of the bright fluorescence observed in live adult animals. Gene-specific probes now available for individual LanFPs will help to establish the identity of green and red emitters at the various stages of lancelet development.

Phylogenetic inference and analysis of conserved intron positions allow us to tentatively place a GFP-like protein into the common ancestor of metazoan animals. The relationship between GFPs and structurally similar G2 domains, however, remains unclear. The similarity between the spatial structures of representative of the two families makes them amenable to structure-based superimposition, and phylogenetic trees based on such alignments have been presented [13]. These trees, however, do not prove the fact of sequence homology in the first place. Using HHpred, which is one of the most sensitive sequence comparison programs and does not rely explicitly on spatial information [15] and the sequence model of G2 domain family derived from pfam07474, we observed several matches to cnidarian fluorescent proteins, with the borderline HHpred P-values from 0.0017 to 0.05. If this borderline sequence similarity, with additional consideration of the same number of β-strands in two families, is taken as the indication of their common ancestry, then the origin of nidogen G2 domain appears to postdate the emergence of GFPs, as G2 domain homologs are detected in nematodes and insects but do not appear in primitive metazoans, such as nearly-completely sequenced cnidarian *N. vectensis *and extensively covered flatworm *Schmidtea mediterranea*. G2 domains are also more diverse in sequence than the GFP-like proteins. If the former have evolved from the latter, the constraints on the sequence of a GFP-like protein, which has to enable the maturation of a chromophore or a fluorophore, have apparently been lifted in the G2 domains, which became coopted into a mutidomain polypeptide that mediates protein-protein interactions in the basal membrane of metazoa. Alternatively, GFP and G2 families may share a common β-barrel ancestor and may have other, still unrecognized relatives in present-day organisms.

Biological functions of GFPs remain elusive. It has been speculated that GFPs in cnidaria may be involved in quenching the UV irradiation [16] and/or in camouflaging coloration against predators [17]. Both hypotheses are compatible with the fact the zone of the brightest fluorescence in lancelets corresponds to the anterior part of the adult animal that is exposed during feeding. On the other hand, lancelets feed on plankton organisms, which display phototaxis, and it is possible that the signal emitted by these proteins serves as bait. On a more practical note, the study of lancelet ESTs and live imaging of animals expands the repertoire of GFP-like proteins, potential sensors and reporters for biological experimentation.

## Methods

### Animal husbandry

Adult lancelets (*B. floridae*) were collected by Marine Biological Supply (Panacea, Florida) at a depth of 10–12 ft at the GPS coordinates N29 degrees, 55.454'/W084 degrees, 15.676' (Apalachee Bay, Wakulla County, FL). They were housed in glass aquariums at pH 8.2–8.4, salinity 33–34 ppt, water temperature 18–19°C, negligible levels of ammonia, nitrite and nitrate, copper and phosphate, on a substrate of either washed, screened play sand or glass beads. A 10% water change was performed once a week. A mix of liquid planktonic foods available commercially for filter feeders was fed daily via a dosing pump. Animals were housed in a room with a lighting level of 30 ft. candles and a 12:12 photoperiod.

### Microscopy

Images were collected using a Zeiss LSM-510 confocal head mounted on a Zeiss axiovert 200 m microscope with AIM acquisition software (Carl Zeiss, Thornwood, USA), coupled to a Chameleon Ultra II IR laser source (Coherent, San Jose) for two photon excitation. Spectral information was acquired using the Meta channel on the Zeiss LSM-510 set to 10 nm resolution with 488 nm excitation. Single channel green image were collected using a confocal microscope equipped with a laser with 488 excitation filter and a 500 to 530 band pass emission filter. Images were acquired with a water immersion objective, 20 × 0.8 NA c-apochromat for the whole animals or 40 × 1.2 NA c-apochromat for cell cultures. For live imaging experiments, animals were mounted in a 25 by 60 mm Nunc chamber with a number 1.5 coverslip. To minimize animal movement, lancelets were anesthetized with buffered MS-222 solution (Tricaine Methanesulfonate) at a concentration of 350 mg/L.

### Spectroscopy

Absorption spectra for purified proteins were obtained using a SOFTmax UV/VIS spectrometer (Molecular Devices). Emission spectra were obtained using a Horiba/Jovan fluoromax fluorescence spectrometer. Emission scans were recorded by exciting the sample at lmax for absorption with 5 nm slit. Molecular absorptivity and quantum yield were measured as in [18] and [19]. The protein concentration for absorbivity measurements was determined by BCA assay and by fluorescence correlation spectroscopy [20].

### Bioinformatics

Sequence similarity searches were performed using the gapped BLAST and PSI-BLAST family of programs [21]. Multiple sequence alignments were done using the MUSCLE program [22]. The JTT distance matrix of protein sequences was constructed using the PROTDIST program and the neighbour-joining phylogenetic tree was constructed using the NEIGHBOR program of the Phylip package [23]. The statistical support of the nodes was assessed by making 100 bootstrap replicates of the aligned sequences, building 100 trees, making the consensus tree, and marking all nodes in the original tree that were supported by more than 50% of the bootstrapped replicates. HMM-to-HMM matching was done using the HHpred server [15].

### Cloning and expression of LanFP1 and LanFP2

LanFP1 and LanFP2 coding sequences have been initially assembled from the *B. floridana *EST libraries as two of the proteins supported by the largest number of EST. Prior to the release of the lancelet genome assembly, there were six nucleotide positions in the LanFP1 coding sequence showing variations between the ESTs in the database, and we used the most frequently conserved nucleotide for these positions. The sequences were subsequently verified by comparing them to the genome assembly. To optimize the codons for expressing in mouse and other mammalian systems, we reverse translated the LanFP1 and LanFP2 into DNA using a standard mouse codon set. For ease of cloning, we included HindIII and BamHI restriction sites at 5' and 3' ends of both sequences. The gene was synthesized by Bioclone, Inc. (San Diego, CA). The synthesized gene was cloned into Sigma's p3XFlag-Myc-CMV plasmid HindIII and BamHI sites, which contained a 5' Flag and a 3' Myc tags plus a stop codon on the vector backbone. The sequence for optimized sequences can be found in Additional File 3.

### Cell Culture and Transfection

Human embryonic kidney (HEK) 293 cells were cultured in the minimum essential medium (MEM) (Invitrogen) supplemented with 5% fetal bovine serum (FBS) and 2 mM glutamine and were maintained at 37°C in a humidified environment of 5% CO2. The cells were plated on 25 mm round coverslips coated with poly-D Lysine (Sigma) 24 to 48 hours and transfected with DNA plasmids when cells reached 70–80% confluence. Plasmid DNA used for transfection was obtained using the HiSpeed maxi-prep kit (Qiagen) and repurified by sodium acetate and ethanol precipitation. 2 μg of plasmid DNA was mixed with 12 μg of Nupherin (Biomol Research Laboratories, Plymouth Meeting, PA) in 300 μl of MEM containing no FBS or antibiotics for 15 min and then combined with 300 μl of MEM containing 6 μl of LipofectAMINE 2000 (Invitrogen) for another 15 min at room temperature. The culture medium was replaced with 600 μl of transfection medium containing the LipofectAMINE-Nupherin-DNA complex. After incubating for 0.5 to 1 hour, the transfection medium was replaced with 2 ml of culture medium. Cell imaging occurred 24–72 hours post transfection.

### Protein purification

BL-21 Ecoli cells were transformed with LanFP1 or LanFP2 subcloned into pRSET-B bacteria expression vector (Invitrogen, Carlsbad, CA) in frame with 6 histidine tags. Overnight cultures were grown to OD 0.4 prior to induction. Bacterial cultures were induced by the addition 1 mM IPTG, grown to OD 0.8, lysed and the His-tagged protein was extracted using a Ni-agarose beads solution (Qiagen cat no. 30210). Concentration of purified protein was established using a BCA assay (Pierce, cat no 23225) and fluorescence correlation spectroscopy.

## List of abbreviations

GFP: Green fluorescent protein.

## Competing interests

The authors have a financial interest in patent applications which cover some of the work described in the paper.

## Authors' contributions

The order of authors is alphabetical. AM, JS and CRY designed research and wrote the manuscript. All authors performed research, analyzed the data, read and approved the final manuscript.

## Reviewers' comments

### Reviewer 1: S. Sunyaev

This manuscript describes a discovery of a family of GFP-like proteins in the newly sequenced genome of a lancelet *B. floridae*. The authors expressed two proteins and detected fluorescent signals of different brightness and wavelengths. Phylogenetic analysis that included the analysis of intron position conservation suggested an early metazoan origin of GFPs followed by a massive parallel loss in multiple lineages. The manuscript also discusses the potential evolutionary relationship between structurally similar G2 domains and GFPs.

### Reviewer 2: M. Matz (nominated by I. King Jordan)

Unfortunately, the novelty edge has been taken away from this work by the prior note by Deheyn et al. of green fluorescent proteins in three species of lancelets (Biol. Bull. 213: 95–100, 2007). Deheyn et al already discussed the phylogenetic relationships of one of the lancelet GFPs, including its relationships with G2 domain proteins, and described *in vivo *fluorescence of lancelets. Still, this paper contains substantially more material than the short note by Deheyn et al. First, the whole family of GFP-like proteins in one genome was surveyed. Second, two of the 12 detected genes have been heterologously expressed and spectroscopically characterized. Third, and this is the most intriguing information for me, red fluorescence has been observed in the lancelet. All these are valuable contributions to our knowledge of diversity and evolution of GFP-like proteins.

I regret, however, that the authors did not go as far as to express and characterize all 12 family members found in the lancelet genome. This would have been a logically complete study, and the first one to describe the full complement of GFP-like proteins from an organism.

*Authors' response*: We are doing it now with all *B. floridae *family members and, for good measure, with the homologs from sea anemone *Nematostella vectensis *that have been revealed in the genome project but, as far as we know, were not characterized before.

In addition to possible identification of the red fluorescent protein, such study might have turned up interesting patterns related to evolution and/or deterioration of gene functions in multigene families. In this regard, it is really intriguing that the two characterized proteins differ so much in their spectral properties. Whereas LanFP1 is a rather generic GFP, LanFP2 is dimmer because of its low quantum yield but instead possesses impressively high molar extinction coefficient, on par with anthozoan purple-blue chromoproteins that are known for their striking color appearance. I would mention in the discussion that this difference in spectral properties may be the result of functional sub-specialization that maintained both genes in the lancelet genome after duplication. Also, given its very high molar extinction, I would expect that LanGFP2 protein should look quite colorful (not necessarily green!) in solution. Is that so?

*Authors' response*: Yes! LanFP2 solution is yellow-brown.

I am rather surprised that the authors doubt the homology of G2 domains and GFPs. In addition to the fold similarity and same collection of the secondary structure elements, these proteins also have the elements in identical (and far from trivial) order within their primary structure. Importantly, this order does not have to be conserved for the protein to work, as has been demonstrated by successful circular permutation of several GFP-like proteins. I believe therefore that in this case, complete identity of the fold including the composition and order of secondary structure elements constitutes a far stronger evidence of homology than any structure-oblivious algorithm may provide.

*Authors' response*: Unfortunately, there is no accepted quantitative model of structural similarity that can be used for testing evolutionary hypotheses in the absence of sequence signal – similar number and similar topology of strands is certainly something worth knowing about, but this qualitative argument cannot (yet) be translated in a hard number, the way the "structure-oblivious" sequence similarities can be. On the other hand, we have nothing against the suggestion that nidogen G2 domain and GFP share a common ancestor. In fact, the relevant paragraph in the Conclusions section includes evolutionary scenarios that presume the existence of just such an ancestor, on the basis of exactly the evidence that the Reviewer highlights. Note also related comments by L. Aravind.

I also have a few minor questions, listed below.

1. How was the fluorescence measured in live animals?

*Authors' response*: The fluorescence was measured using a LSM-510 confocal microscope. The animals were immobilized with a muscle inhibitor and images collected using 488 nm laser excitation and spectral imaging with either a 20× or 40× objective (see Methods for more detail).

2. What were the excitation wavelengths for "green" and "red spectra", Fig. 2E?

*Authors' response*: The excitation wavelength was 488 nm with an argon laser for both green and red spectra. The red spectrum can also be excited with a 561 nm laser. The two-photon excitation scan indicates the green fluorescence is produced when excited with 720 to 1020 nm light. The red fluorescence was not apparent until using two-photon excitation above 980 nm light.

3. The "red spectra" does not look like any of the GFP-like proteins known thus far. What can you say about it?

*Authors' response*: The spectrum does not provide any information about the potential source of the red fluorescence, i.e., even whether it is a protein or a small molecule. Note, however, the claims in a very recent patent (ref. 14) about proteins from a related species of *Branchiostoma*.

4. I can't help noticing that the "green spectra" are different in Fig. 2E and 2F, although they are supposed to be the same. Why?

*Authors' response*: They are identical. We hope that reformatted figure makes this clearer.

5. I'd like to see excitation spectra for the expressed proteins (augment Fig. 2F).

*Authors' response*: These data are presented in the added Supplementary Figure S4 (Additional File 4). They were collected using fluorescence spectrometer with 1 nm resolution (i.e., higher than the 10 nm-resolved spectra obtained on LSM-510).

6. Does lancelet genome contain G2 domains? If not, please discuss that, if yes, please put them into the phylogeny.

*Authors' response*: Yes, it does. These sequences are solidly within the chordate clade, not interminglingmingling with deeper-branching invertebrate homologs in the same tree. Thus, this domain is clearly distinct from GFP domain, and adding G2 sequences to the tree would not help to better illustrate or to refute any point that we are trying to make in this article: in order to include a sequence into the alignment that is used for evolutionary inference, homology needs to be proven already (see response on the monophyly question above and also to L. Aravind comment below).

7. Please make the phylogenetic tree one of main figures. I would also appreciate if the tree was reformatted into a more conventional form.

*Authors' response*: We feel that the tree is too trivial to add much to discussion (in fact, except for the addition of more *Branchiostoma *and *Nematostella *sequences, it is nearly identical to the tree shown in ref. 1), but we now provide, as Additional File 5, two trees in Newick format: 1. the main neighbour-joining tree, which contains branch length information, and 2. the consensus of 100 neighbour-joining trees built on the basis of the bootstrap replicates of the first tree, which contains the information on the bootstrap support for each partition in the first tree.

8. The role of fluorescence in attracting food is an interesting proposition, but why it is important particularly during twilight? I don't see the connection.

*Authors' response*: Phytoplankton and zooplankton exhibit phototaxis, and different parts of spectra may be most effective at different times of day and at different depths in attracting food from the surface. We agree that there is no particular evidence that would compel one to focus on twilight, so we deleted "during twilight".

### Reviewer 3: L. Aravind

The paper by Baumann et al describes the recovery of several new GFP related proteins in amphioxus (*B. floridae*) and experimental characterization of a few of them. The point of note is the proposal that that GFP was present in the common ancestor of *Bilateralia *and *Cnidaria *and repeatedly lost in various animal lineages. Despite the extremely sporadic distribution of these proteins, the phylogenetic analysis and the argument of conserved intron positions support this scenario. Yet, it might be useful to check environmental metagenomic sequences to address the possibility of independent lateral transfer from some unknown source into the few animal lineages in which GFPs are found.

*Authors' response*: We searched metagenomic sequences, but found only the garden-variety GFPs closely identical to the founder from *A. victoria*, only in marine samples and represented by small number of collapsed reads. We think they are encoded by hydroid DNA present in some of these samples.

Even more striking is the proposal that the nidogen G2 domains have emerged from the GFP like family after the divergence of cnidarians and possibly flatworms from the remaining metazoans. This proposal is weakly supported. Given that fact that sequence similarity between the G2 domain and GFP is only detected using the most sensitive profile-profile comparison method, it is entirely possible that there remain undetected β-barrel proteins with comparable topologies and geometry that are the precursors of both GFP and G2 domains. While this may sound less parsimonious, the high levels of sequence divergence observed in various β barrel folds (especially when there no comparable functional constraints) do make it a distinctive possibility.

*Authors' response*: We agree that this is a possibility worth discussing, and we amended the text accordingly. Note, however, that we have not seen any G2 domains in complete proteome of sea anemone *Nematostella *or in the available sequences of planarian *Schmidtea *or any other primitive metazoan clade. Thus, if the common 11-stranded barrel ancestor of G2 domain and of the present-day GFPs was not itself part of the GFP family, it either had to spawn GFP and G2 domains on separate occasions, or to split into GFP and G2 followed by at least one, or possibly many losses, of G2 (in *Anthozoa *and perhaps in *Plathelminthes*). Better sequence sampling of sponges, coelenterates and flatworms would help to sort out these possibilities.

Minor points: -Pg 10 "At this level of significance, GFPs did not stand out from the background of other β-barrel proteins." What were the other β barrel proteins detected by HHpred and their p-values? Was a reciprocal search done using the GFP profiles as queries against a profile database lacking GFPs?

*Authors' response*: This paragraph was a bit too convoluted in the initial version. Current HHPred server (release 2.2.1) does not report GFP-G2 similarity if the complete database is used as the search space. The same negative results are obtained with the stand-alone version 1.5.0 whether or not the selfsame models are removed from the database. The matches between G2 and GFP can be only observed when we search the subset of HMMs built upon proteins with the known three-dimensional structures. In that case, the G2 model finds the best GFP model with p-value 0.0017, and other GFP-related models have p-values from 0.002 to 0.005. An apparently unrelated β-barrel family, specified by At1g79260 protein from *Arabidopsis thaliana*, is seen with p-value 0.039, though the alignment is only partial and reciprocal search does not detect the G2 domain. G2 model also finds bacterial semi-barrel LolB in searches against the complete database (p-value 0.022: interestingly, LolB also has 11 strands). GFP does not detect G2 even under these forced conditions. All these weak similarities, however, only reinforce our main argument, i.e., that we still do not have a robust statistical validation of the GFP-G2 monophyly at the sequence level. Thus, interested as we are in discussing scenarios that assume a common ancestor, we can do it only hypothetically. We have modified the Conclusions section with this in mind.

What is the evidence that the red fluorescence comes from a GFP like protein and not some other fluorochrome?

*Authors' response*: This question was also raised by M. Matz, see our response (the short answer is: no evidence, but see ref. 14).

## Supplementary Material

Additional file 1 Phylogenetic tree of GFP-like proteins. The image of the neighbour-joining tree inferred from the alignment of GFP-like proteins. Click here for file

Additional file 2 Spectral properties of LanFP2 and LanFP2. The table summarizing absorbance, emission, extension and quantum yield for several GFP-like proteins Click here for file

Additional file 3 Murinized sequences of LanFP1 and LanFP2. Nucleotide sequences encoding two *B. floridae *fluorescent proteins, with codon usage optimized for expression in murine cells Click here for file

Additional file 4 Normalized absorbance spectra of LanFP1 and LanFP2. Image of spectra of two *B. floridae *fluorescent proteins compared with several other spectra of GFP-like proteins Click here for file

Additional file 5 Source file of the tree shown in Additional file 1. Newick-formatted trees, one of which contains branch length information, and the other is the consensus of 100 trees built on the basis of the bootstrap replicates of the first tree, contains the information on the bootstrap support for each partition in the first tree but lacking the branch length information. Click here for file
