# Supplementary material for: A family of GFP-like proteins with different spectral properties in lancelet Branchiostoma floridae
Source: Biol Direct. 2008 Jul 3;3:28. doi: 10.1186/1745-6150-3-28 (PMC2467403; doi:10.1186/1745-6150-3-28)
Supplement: Additional file 1 — Phylogenetic tree of GFP-like proteins. The image of the neighbour-joining tree inferred from the alignment of GFP-like proteins. [file 1745-6150-3-28-S1.doc]

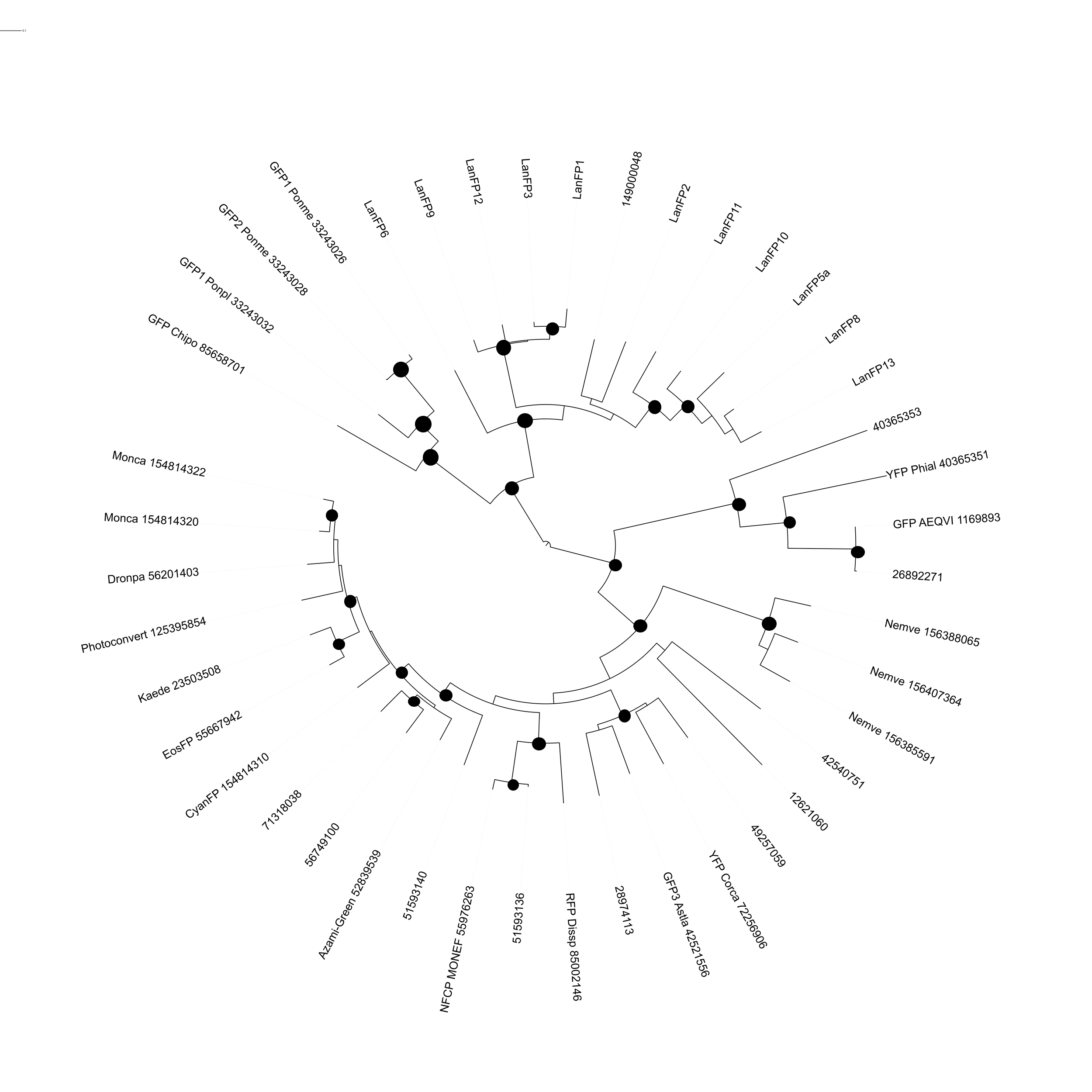


Figure S1. The neighbor-joining tree of representative GFP-like sequences from cephalochords, copepods and cnidarians. Internal nodes with bootstrap support higher than 80% are indicated by large dots.
