# Supplementary material for: A family of GFP-like proteins with different spectral properties in lancelet Branchiostoma floridae
Source: Biol Direct. 2008 Jul 3;3:28. doi: 10.1186/1745-6150-3-28 (PMC2467403; doi:10.1186/1745-6150-3-28)
Supplement: Additional file 2 — Spectral properties of LanFP2 and LanFP2. The table summarizing absorbance, emission, extension and quantum yield for several GFP-like proteins [file 1745-6150-3-28-S2.pdf]

	λmax Absorbance	λmax Emision	Molar Extinction $M^{-1}cm^{-1}$	QY	Relative Brightness
**GFP**	490	510	32000	76.16	1.00
**YFP**	516	528	70000	73.38	2.11
**LanFP1**	500	510	53000	57	1.02
**LanFP2**	500	516	162000	0.13	0.01
