# Supplementary material for: A family of GFP-like proteins with different spectral properties in lancelet Branchiostoma floridae
Source: Biol Direct. 2008 Jul 3;3:28. doi: 10.1186/1745-6150-3-28 (PMC2467403; doi:10.1186/1745-6150-3-28)
Supplement: Additional file 3 — Murinized sequences of LanFP1 and LanFP2. Nucleotide sequences encoding two B. floridae fluorescent proteins, with codon usage optimized for expression in murine cells [file 1745-6150-3-28-S3.doc]

Murinized coding sequences for LanFP1 and 2:

LanFP1

atgcccctgcccgccacccacgacatccacctgcacggcagcatcaacggccacgagttcgacatggtgggcggcggcaagggcgaccccaacgccggcagcctggtgaccaccgccaagagcaccaagggcgccctgaagttcagcccctacctgatgatcccccacctgggctacggctactaccagtacctgccctaccccgacggccccagccccttccaggccagcatgctggagggcagcggctacgccgtgtaccgcgtgttcgacttcgaggacggcggcaagctgaccaccgagttcaagtacagctacgagggcagccacatcaaggccgacatgaagctgatgggcagcggcttccccgacgacggccccgtgatgaccagccagatcgtggaccaggacggctgcgtgagcaagaagacctacctgaacaacaacaccatcgtggacagcttcgactggagctacaacctgcagaacggcaagcgctaccgcgcccgcgtgagcagccactacatcttcgacaagcccttcagcgccgacctgatgaagaagcagcccgtgttcgtgtaccgcaagtgccacgtgaaggccagcaagaccgaggtgaccctggacgagcgcgagaaggccttctacgagctggccgga

lanFP2

atgagcctgcccaccacccacgacctgcacatcttcggcagcgtgaacggcgccgagttcgacctggtgggcggcggcaagggcaaccccaacgacggcaccctggagaccagcgtgaagagcacccgcggcgccctgccctgcagccccctgctgatcggccccaacctgggctacggcttctaccagtacctgcccttccccggcggcgccagccccttccagaccgccatcaccgacggcggctaccaggtgcaccgcgtgttcaagttcgaggacggcggcgtgctgagctgcaacttccgctacacctacgagggcggcaagatcaagggcgagttccagctgatcggcagcggcttccccgccggcggccccgtgatgagcggcggcctgaccaccctggaccgcagcgtggccaagctgcagtgcagcgacgactgcaccatcaccggcaccaacaactggagcttctgcaccaccgacggcaagcgccaccaggccgacgtgcagaccaactacaccttcgccaagcccctgcccgccggcctgaaggagaagatgcccatcttcctgggccaccagatcgaggtgaaggccagcaagaccgagatcaccctgagcgagaaggtgaaggccttcatcgacaccgtgggcagcggtaccgga
