# Supplementary figures and images for: A family of GFP-like proteins with different spectral properties in lancelet Branchiostoma floridae
Source: Biol Direct. 2008 Jul 3;3:28. doi: 10.1186/1745-6150-3-28 (PMC2467403; doi:10.1186/1745-6150-3-28)

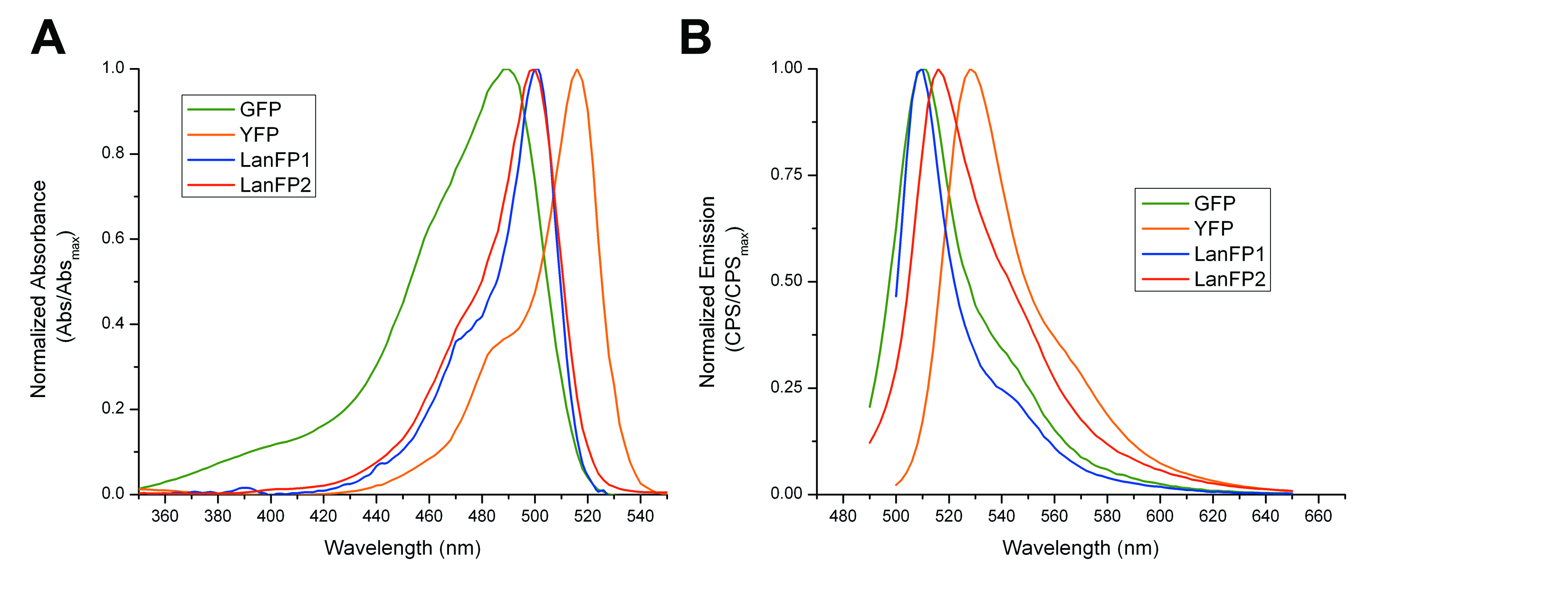

Supplement: Additional file 4 — Normalized absorbance spectra of LanFP1 and LanFP2. Image of spectra of two B. floridae fluorescent proteins compared with several other spectra of GFP-like proteins [file 1745-6150-3-28-S4.jpeg]
